# Orthodontic Visits According to Socioeconomic Status Among Children Living in the City of Kuopio, Finland: The PANIC Study

**DOI:** 10.1002/cre2.70280

**Published:** 2026-01-09

**Authors:** Maria Viisanen, Eero Raittio, Tiina Ikävalko, Timo Peltomäki, Anna Liisa Suominen, Ville Tolonen, Sonja Soininen, Henri Karvinen, Timo A. Lakka

**Affiliations:** ^1^ Oral Health Care Wellbeing Services County of Central Finland Jyväskylä Finland; ^2^ Department of Dentistry and Oral Health Aarhus University Aarhus Denmark; ^3^ Institute of Dentistry University of Eastern Finland Kuopio Finland; ^4^ Odontology Education Unit Wellbeing Services County of North Savo Kuopio Finland; ^5^ Faculty of Medicine and Health Technology Tampere University Tampere Finland; ^6^ Department of Ear and Oral Diseases Tampere University Hospital Tampere Finland; ^7^ Oral Health Care Wellbeing Services County of North Ostrobothnia Oulu Finland; ^8^ Institute of Biomedicine University of Eastern Finland Kuopio Finland; ^9^ Teaching Clinic Osmo Wellbeing Services County of North Savo Kuopio Finland; ^10^ Institute of Public Health and Clinical Nutrition University of Eastern Finland Kuopio Finland; ^11^ Department of Clinical Physiology and Nuclear Medicine Kuopio University Hospital Kuopio Finland; ^12^ Foundation for Research in Health Exercise and Nutrition Kuopio Research Institute of Exercise Medicine Kuopio Finland

**Keywords:** dental care for children, healthcare disparities, orthodontics, parents, universal health insurance

## Abstract

**Objective:**

The aim of the present study was to examine whether the parental socioeconomic status of children was related to the number of orthodontic visits or orthodontic care from birth to adolescence.

**Material and Methods:**

The analyses are based on data from the Physical Activity and Nutrition in Children (PANIC) study in a population sample of 504 children aged 7–9 years at baseline in 2007–2009. Parental education and household income at baseline were used as indicators of parental socioeconomic status. The number of orthodontic visits from birth to the age of 16 years was obtained from local healthcare registers.

**Results:**

On average, the participants had 16.1 (standard deviation [SD] 15.6) orthodontic visits during the follow‐up period of 15.9 (SD 0.72) years. Of the participants, 58% had received orthodontic care, defined as at least six orthodontic visits including at least one orthodontic procedure and not just screening visits, and they had, on average, 25.8 (SD 14.1) orthodontic visits over the follow‐up. Neither parental education nor household income was associated with the number of orthodontic visits or receiving orthodontic care during the follow‐up. For instance, compared to low parental education and income groups, high education (−2.86; 95% confidence interval: −6.75; 1.03) and income groups (−0.08; 95% confidence interval: −4.66; 4.51) did not have considerably different numbers of orthodontic visits over the follow‐up. Among children from families with lower parental education who received orthodontic care, boys had, on average, 21 visits (95% confidence interval: 16; 26) and girls had 31 visits (95% confidence interval: 25; 36).

**Conclusions:**

Parental socioeconomic status did not seem to affect the number of orthodontic visits or the receipt of orthodontic care among children living in the city of Kuopio, Finland.

**Clinical Trial Registration:**

The Physical Activity and Nutrition in Children (PANIC) Study (PANIC) was registered in ClinicalTrials.gov, NCT01803776

## 1 Introduction

The number of children and adolescents receiving orthodontic care has been found to vary between 7% and 35% depending on the country (Mohlin et al. 2007; Laniado et al. 2017; Linden et al. 2019; Mitchell and Littlewood 2019; Jiang et al. 2024). Orthodontic treatments have been found to account for about 14%–20% of all oral healthcare treatments in children and adolescents, considering the private and public sectors (Laniado et al. 2017; Linden et al. 2019). The practices of different countries regarding access to orthodontic treatments vary depending on the healthcare system, the costs of treatments to the patient, or the availability of resources and personnel (Linden et al. 2019). In addition, the criteria for assessing the need for orthodontic care vary across countries.

Parental socioeconomic status is associated with the general health of children (Mäki and Laatikainen 2012; Capurro et al. 2015). Inequality in the use of dental services is a global challenge, and dental services utilization is lower among less educated people, ethnic minorities, and immigrants, as well as in rural areas (Reda et al. 2018). However, the inequalities in the use of dental services seem to be lower in countries with higher public coverage for oral healthcare than in countries primarily relying on privately covered oral healthcare (Reda et al. 2018). Findings from studies conducted in countries like the United States and the United Kingdom have found that children with lower parental socioeconomic status tend to have fewer orthodontic visits, receive less orthodontic care, and are more likely to discontinue orthodontic care (Laniado et al. 2017; Okunseri et al. 2007; Ulhaq et al. 2012; Berdahl et al. 2016; Ghonmode et al. 2022; Price et al. 2017). On the other hand, studies on the duration of orthodontic care by socioeconomic status have not found considerable differences (Nichols and Fowler 2018; Nakhleh et al. 2020; Lemasney and Mathur 2023). Universal health coverage (UHC) advocated by the World Health Organization emphasizes promoting equity in access to care by access to key promotive, preventive, curative, and rehabilitative health interventions for all at an affordable cost (World Health Assembly 2005). Using the UHC principle in the orthodontic setting would prioritize the need for care over socioeconomic factors as the basis for prioritizing public orthodontic services. Recent findings from Norway also support this view, as they showed that starting orthodontic care in children and adolescents was not associated with household income under an oral healthcare system with high public coverage (Jiang et al. 2024). On the other hand, there seem to be regional, educational, and other social differences in receiving orthodontic care in Norway, though these findings are based on descriptive analyses and do not account for potential confounders, such as adjustments for other socioeconomic variables (Texmon and Ekornrud 2023).

In Finland, oral healthcare, including orthodontic treatments for children and adolescents under 18 years of age, is delivered by public oral healthcare services. In line with the UHC principle, oral healthcare services for children are available at no cost and encompass examinations, preventive, restorative, and orthodontic treatments tailored to each child's needs. According to a previous study, 30% of Finnish children and adolescents have received orthodontic care (Linden et al. 2019; Finnish Institute for Health and Welfare 2024), and orthodontic treatments accounted for 20% of all oral healthcare treatments (Linden et al. 2019). The need for orthodontic care is assessed in the different stages of dental development during childhood and adolescence, using the national 10‐step classification of malocclusions (Heikinheimo 1989), and only the ones with the severest malocclusions are offered treatment (Ministry of Social Affairs and Health 2019). The decision to initiate orthodontic care is made collaboratively between the family and oral healthcare professionals. The aim of the uniform criteria was to harmonize the policies regarding eligibility for orthodontic care in public oral healthcare services, but still, there are different practices due to the financial situation of the counties, the number of specialized dentists or experienced dentists in orthodontics, and the number of children and adolescents living in different areas in Finland. These constraints and variations may result in unwarranted disparities in the allocation of services, potentially influenced by factors such as socioeconomic status.

We have limited knowledge of whether parental socioeconomic status is associated with children's and adolescents' orthodontic care in Finland. The aim of the present study was to examine whether parental socioeconomic status is related to receiving orthodontic care or the number of orthodontic visits among children and adolescents.

## 2 Methods

### 2.1 Study Design and Participants

The present secondary analyses are based on data from the Physical Activity and Nutrition in Children (PANIC) study (ClinicalTrials.gov NCT01803776), which aims to investigate the effects of an individualized and family‐based physical activity and dietary intervention on cardiometabolic risk factors and other health outcomes in a general population of children aged 6–9 years followed up for 8 years until adolescence. Altogether, 736 children aged 7–9 years who started the first grade in 16 primary schools of the city of Kuopio, Finland, in 2007–2009, were invited to the PANIC study. Of all the children invited, 512 children (248 girls and 264 boys), who accounted for 70% of those invited, participated in the baseline examinations in 2007–2009. Based on data from the standard school health examinations performed for all Finnish children before the first grade, the participants did not differ in age, gender, or height—or body mass index—standard deviation (SD) score from those who did not participate. At baseline, 6 children were excluded from the study because of physical disabilities that could hamper participation in the intervention, or a lack of time or motivation to attend the study. Two children whose parents later withdrew their permission to use the data of their children were also excluded. Thus, 504 children (240 girls and 264 boys) were included in the study, and those children have been followed retrospectively since birth in 1999–2001 using register data and prospectively since baseline in 2007–2009 until the end of 2016 (around the age of 16). Sample size calculations in the PANIC study have been explained in detail elsewhere (Lakka et al. 2020), separate sample size calculations for this secondary study were not conducted.

The Research Ethics Committee of the Hospital District of Northern Savo approved the study protocol in 2006 (Statement 69/2006) and in 2015 (422/2015). The caregivers provided informed consent, and the children gave assent to participation at baseline, and the caregivers and the adolescents provided informed consent at the 8‐year follow‐up. The PANIC study has been carried out in accordance with the principles of the Declaration of Helsinki and its amendments.

### 2.2 The Use of Orthodontic Services

For this study, data on the use of oral healthcare services (date of visit and treatment provided) from birth to the end of 2016 among participants in the PANIC study were collected in 2019 by four dental students from local oral healthcare registers in Kuopio. Data from birth onward were included because there is no minimum age for orthodontic screening or treatments in Finland. Visits to specialized or experienced dentists in orthodontics, dental hygienists, or dental nurses concerning orthodontics were included. Other entries in the oral healthcare records not related to actual visits in the healthcare facility, such as canceled visits, no‐shows, phone calls, treatment planning, or consultations, were not counted as visits.

**Figure 1:**
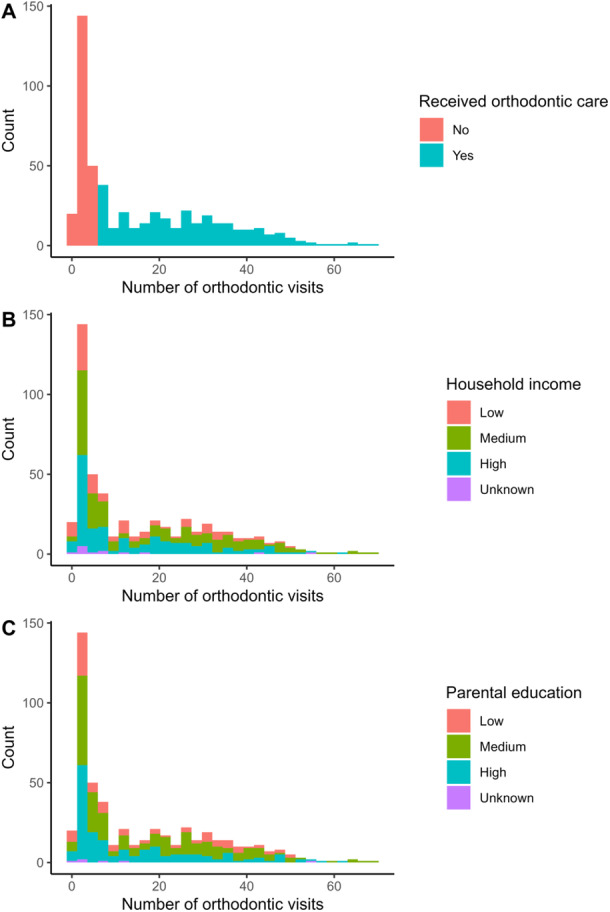
Distribution of the number of orthodontic visits (A) by household income (B) and parental education (C) status from birth until the end of the year 2016. Receiving orthodontic care was defined by having six or more orthodontic visits during the study period.

Based on manual review of the data on orthodontic visits, we found that when there were six or more orthodontic visits during the study period, the child had always received an orthodontic procedure (e.g., appliance placement) and not just screening examinations. Therefore, the child was considered to have received orthodontic care if the child had orthodontic visits of at least six (Figure 1).

### 2.3 Background Information

The parents answered a structured questionnaire at the baseline of the PANIC study in 2007–2009 when the children were 7–9‐year‐olds. Parental education and household income were used as indicators of socioeconomic status. Parental education was defined as the highest completed or ongoing degree of the parents and categorized into three classes (Low: occupational education, Medium: occupational institute or university of applied sciences, and High: university). Parental annual household income without deducting taxes was categorized into three classes (Low: 30,000€ or less, Medium: 30,001–60,000€, and High: 60,001€ or more). The national median household income was around 30,000€ in 2007 (Official Statistics of Finland 2007). Other information used in the statistical analyses included the child's age and gender, mother's marital status (married or in cohabitation; single; separated or divorced; or widow), as well as parental self‐rated health and self‐reported depression or other psychiatric disorders diagnosed by a physician. Parental self‐rated health was categorized as very poor, poor, good, very good, and excellent according to the parent whose self‐rated health was worse. The participants were divided into two groups depending on whether at least one of the parents reported having had depression or another psychiatric disorder.

### 2.4 Statistical Methods

To handle missing data in the background information (Table 1), multiple imputation with chained equations with 93 variables (Supporting Information) was used. The number of imputations (*n* = 10) was determined using the method described by von Hippel (von Hippel 2018). With these 10 imputed datasets, the association between socioeconomic status variables and receiving orthodontic care (yes/no) was investigated with logistic regression, and the results were pooled to obtain final estimates. In the same way, the association with the number of orthodontic visits was investigated with linear regression among all participants and separately among those who had received orthodontic care (i.e., had six or more orthodontic visits).

**Table 1:** Characteristics of participants.

		Received orthodontic care, *N* (%)*			Number of orthodontic visits (mean, SD)			
	Total	No	Yes	*p* value	Total	*p* value	Received orthodontic care*	*p* value
*N*/Total	504	214	290	—	504	—	290	—
Number of orthodontic visits (mean, SD)	16.1 (15.6)	3.0 (1.1)	25.8 (14.1)	—	—	—	—	—
Age at the baseline (mean, SD)	7.6 (0.02)	7.6 (0.03)	7.6 (0.02)	0.73	—	—	—	—
Age at the end of 2016 (mean, SD)	15.9 (0.7)	15.9 (0.7)	15.9 (0.7)	0.53	—	—	—	—
Gender				0.73		0.12		0.04
Girl	240	100 (42)	140 (58)		17.2 (16.1)		27.5 (13.6)	
Boy	264	114 (43)	150 (57)		15.0 (15.1)		24.1 (14.4)	
Household income				0.28		0.01		0.02
Low	106	50 (47)	56 (53)		13.9 (13.7)		23.8 (12.0)	
Medium	204	78 (38)	126 (62)		18.8 (14.1)		28.5 (15.2)	
High	181	79 (44)	102 (56)		14.6 (14.1)		23.6 (12.9)	
Unknown	13	7	6		12.1 (17.2)		23.5 (20.4)	
Parental education				0.26		0.13		0.45
Low	98	40 (41)	58 (59)		16.6 (15.5)		26.2 (13.3)	
Medium	222	87 (39)	135 (61)		17.4 (16.1)		26.6 (14.3)	
High	178	84 (47)	94 (53)		14.3 (14.8)		24.3 (14.1)	
Unknown	6	3	3		13.2 (20.5)		24.7 (25.5)	
Mother's marital status				0.23		0.05		0.13
Married/cohabitation	412	168 (41)	244 (59)		16.9 (16.0)		26.5 (14.3)	
Single	30	13 (43)	17 (57)		14.8 (14.0)		24.1 (11.9)	
Separated/divorced	49	27 (55)	22 (45)		10.8 (11.3)		20.4 (10.8)	
Widow	2	1 (50)	1 (50)		5.5 (5.0)		—	
Unknown	11	5	6		13.1 (16.9)		22.0 (19.0)	
Parental self‐rated health				0.77		0.10		0.02
Excellent	38	16 (42)	22 (58)		20.3 (18.0)		32.9 (13.4)	
Very good	206	90 (44)	116 (56)		15.1 (14.4)		24.4 (12.9)	
Good	174	67 (39)	107 (61)		16.9 (15.9)		25.7 (14.6)	
Poor	53	22 (42)	31 (58)		18.2 (17.1)		28.9 (15.0)	
Very poor	5	3 (60)	2 (40)		4.6 (2.7)		7.5 (0.71)	
Unknown	28	16	12		10.4 (14.4)		21.7 (16.3)	
Parental psychiatric disorder				0.08		0.18		0.96
Yes	88	45 (51)	43 (49)		14.1 (15.1)		25.9 (14.0)	
No	407	166 (41)	241 (59)		16.5 (15.6)		25.7 (14.2)	
Unknown	9	3	6		15.3 (18.1)		22.0 (19.0)	

*Note: p* values from ANOVA.

* Received orthodontic care (6 visits or more) and the number of orthodontic visits from birth until the end of the year 2016.

Based on assumptions about causal relationships among the available variables, a directed acyclic graph was constructed to identify confounders that influence both socioeconomic status and receiving orthodontic care, as well as precision variables that only affect receiving orthodontic care (Figures S1 and S2). Considering previous literature on socioeconomic inequalities in (oral) health and healthcare (Capurro et al. 2015; Reda et al. 2018; Ghonmode et al. 2022), we hypothesize that parental educational level and household income are associated with orthodontic care use. We assumed that a child's gender and date of birth (age) are unaffected by parental educational level or household income but might influence the use of orthodontic services (i.e., they are precision variables). Additionally, we assumed that parental education, mother's marital status, parental self‐rated health, and self‐reported depression or other psychiatric disorders could potentially affect both household income and orthodontic service use (i.e., they are confounders). Parental self‐rated health and self‐reported depression or other psychiatric disorders were also assumed to be confounders of the association between parental educational level and orthodontic service use.

Taking these assumed causal relationships into account, three models that were differently adjusted for confounding and precision variables were used to investigate the association between socioeconomic status variables and orthodontic care. The first model included only parental education or income, the second model included confounders, and the third model included confounders and precision variables (Table 2). Additionally, we tested the interaction between socioeconomic status variables and gender by including their interaction terms in the fully adjusted models. Statistical analyses were performed using R (v 4.3.1, R Foundation for Statistical Computing, Vienna, Austria).

**Table 2:** Association of parental education and household income with receiving orthodontic care and number of visits using 10 multiple imputed datasets.

**Outcome: Received orthodontic care (6 or more visits, yes/no), odds ratio, and 95% confidence interval, *N*** = **504.**				
	Proportion (SE)	Model I	Model II	Model III
Education (ref. Low)	0.59 (0.01)	1.00	1.00	1.00
Education: Medium	0.61 (0.01)	1.08 (0.67; 1.75)	0.99 (0.60; 1.62)	0.99 (0.60; 1.62)
Education: High	0.53 (0.01)	0.78 (0.48; 1.28)	0.71 (0.43; 1.19)	0.71 (0.42; 1.19)
Income (ref. Low)	0.53 (0.02)	1.00	1.00	1.00
Income Medium	0.61 (0.01)	1.44 (0.90; 2.30)	1.42 (0.85; 2.37)	1.42 (0.86; 2.37)
Income High	0.56 (0.01)	1.17 (0.73; 1.88)	1.32 (0.75; 2.32)	1.32 (0.75; 2.31)

**Outcome: Number of orthodontic visits among all participants**, **regression coefficients, and 95% confidence intervals, *N*** = **504.**				
	Mean (SE)	Model I	Model II	Model III
Education (ref. Low)	16.4 (0.49)			
Education: Medium	17.4 (0.34)	0.97 (−2.66; 4.61)	0.46 (−3.26; 4.19)	0.26 (−3.47; 4.00)
Education: High	14.3 (0.35)	−2.16 (−5.93; 1.62)	−2.91 (−6.80; 0.97)	−2.86 (−6.75; 1.03)
*R* ^2^		0.01	0.03	0.03
Income (ref. Low)	14.0 (0.42)			
Income Medium	18.7 (0.38)	4.71 (1.10; 8.31)	3.14 (−1.00; 7.28)	3.16 (−0.98; 7.31)
Income High	14.5 (0.33)	0.49 (−3.20; 4.14)	−0.16 (−4.74; 4.43)	−0.08 (−4.66; 4.51)
*R* ^2^		0.02	0.05	0.05

**Outcome: Number of orthodontic visits among those in orthodontic care (had 6 or more visits)**, **regression coefficients, and 95% confidence intervals, *N*** = **290.**				
	Mean (SE)	Model I	Model II	Model III
Education (ref. Low)	26.1 (0.55)			
Education: Medium	26.6 (0.39)	0.52 (−3.77; 4.81)	0.73 (−3.58; 5.05)	0.35 (−4.01; 4.72)
Education: High	24.3 (0.46)	−1.82 (−6.39; 2.76)	−2.31 (−6.98; 2.36)	−2.23 (−6.90; 2.44)
*R* ^2^		0.01	0.04	0.05
Income (ref. Low)	24.2 (0.55)			
Income Medium	28.4 (0.43)	4.26 (−0.11; 8.63)	3.90 (−0.90; 8.69)	3.68 (−1.16; 8.50)
Income High	23.4 (0.40)	−0.75 (−5.26; 3.75)	−0.12 (−5.48; 5.23)	−0.02 (−5.37; 5.34)
*R* ^2^		0.03	0.06	0.07

*Note:* Adjustment sets for parental education, Model I: only parental education; Model II: Model I + parental self‐rated health + parental psychiatric disorder; Model III: Model II + age + gender. Adjustment sets for household income, Model I: only household income; Model II: Model I + parental education + parental self‐rated health + parental psychiatric disorder + mother's marital status; Model III: Model II + age + gender.

## 3 Results

The characteristics of 504 children and adolescents are described in Tables 1 and S1. More than half (*n* = 290, 57.5%) of the participants had 6 or more orthodontic visits and thus received orthodontic care during the follow‐up period (Table 1 and Figure 1), accounting for 92.2% (7473) of all 8105 orthodontic visits. Girls and boys who received orthodontic care were, on average, of the same age at the baseline, 7.6 (SD 0.4) and 7.7 (SD 0.4), respectively (Tables 1 and S1). Among girls, most of the participants who received orthodontic care were in the medium income (44%) and education (51%) groups, whereas among boys, most were in the highest income (41%) and education (41%) groups (Table S1).

On average, the participants had 16.1 (SD 15.6) orthodontic visits over the follow‐up period of 16 years, with visits taking place between the years 2001 and 2016 (Table 1 and Figure 1). Those who had received orthodontic care had an average of 25.8 (SD 14.1) orthodontic visits over the 16 years (Table 1 and Figure 1). The number of no‐show orthodontic visits was relatively low (*n* = 287, 3.5% compared to all orthodontic visits), and orthodontic care was discontinued in 19 (of 290, 6.6%) children during the follow‐up.

No statistically significant differences in receiving orthodontic care during the follow‐up period between the three education or income groups were found, regardless of adjustments (Table 2).

Among all participants, children in the middle household income group had, on average, 4.7 (95% confidence interval: 1.1; 8.3) orthodontic visits more than children in the lowest household income group in the unadjusted analyses, but in the adjusted models, the association was weaker and not statistically significant (Table 2). Otherwise, neither income nor education was associated with the number of orthodontic visits over the follow‐up.

Among girls, medium household income was significantly associated with a higher probability of receiving orthodontic care and a higher total number of orthodontic visits compared to girls from lower and higher income households (Figure S3A,C). Whereas, for boys, no differences were observed in the probability of receiving orthodontic care or the total number of orthodontic visits between socioeconomic groups.

Among those who received orthodontic care, there were no statistically significant differences in the number of orthodontic visits during the follow‐up period across the three parental education or household income groups with any adjustments (Table 2). Among those who received orthodontic care, boys from lower parental education families had, on average, 21 visits (95% confidence interval: 16; 26) and girls had 31 visits (95% confidence interval: 25; 36) during the follow‐up (Figure S3F). Otherwise, there were no differences in the number of visits by gender among those who received orthodontic care.

## 4 Discussion

Children had, on average, 16 orthodontic visits from birth to the age of 16 years, and those who received orthodontic care, representing 58% of participants, had, on average, 26 orthodontic visits. The findings of the present study suggest that family socioeconomic status does not affect the number of orthodontic visits or the receipt of orthodontic care from birth to adolescence. Household income influenced the outcomes differently by gender, with a medium household income being associated with a higher probability of receiving orthodontic care and a higher total number of orthodontic visits among girls, but not among boys. No consistent differences were observed by parental education, except that among those who received orthodontic care, boys from lower parental education families had, on average, 10 fewer visits than girls from lower parental education families.

Previous studies, conducted, for example, in the United States or the United Kingdom, have found that children living in lower socioeconomic conditions have fewer orthodontic visits and receive less orthodontic care than those at better socioeconomic conditions (Laniado et al. 2017; Okunseri et al. 2007; Ulhaq et al. 2012; Berdahl et al. 2016; Ghonmode et al. 2022; Lemasney and Mathur 2023). In contrast, our findings and those from studies conducted in other Nordic countries have indicated lower inequalities, which can be expected when oral health systems reflect the principles of UHC, as Nordic oral healthcare systems for children and adolescents do. A recent Norwegian study found that parental income was not associated with receiving orthodontic care (Jiang et al. 2024), which aligns with the present findings, as parental socioeconomic status, as indicated by parental education and household income, was not associated with the number of orthodontic visits or receiving orthodontic care. According to the results of an older Danish study among children and adolescents aged 11–15 years, those from lower socioeconomic groups had higher rates of discontinuing orthodontic treatments compared to those from higher socioeconomic groups, yet no considerable difference in receiving orthodontic treatments was found between socioeconomic groups (Rölling 1982). To our knowledge, no research data are available regarding the impact of socioeconomic status on orthodontic care in Sweden or Iceland.

In general, orthodontic treatment is characterized by several visits, which require commitment and motivation from the whole family, not just from the child or adolescent. For this reason, we would expect to see some differences by socioeconomic status, because they are generally associated with, for example, health literacy, commitment, or motivation for health‐related issues. Accordingly, it has been observed that the mother's emotional support and encouragement to the child are associated with shorter treatment duration and success of orthodontic care (Nakhleh et al. 2020; Joury et al. 2013), even when taking socioeconomic status into account (Joury et al. 2013). Therefore, further studies could focus on identifying patient and family characteristics and conditions that are more strongly associated with challenges in receiving orthodontic care than socioeconomic status, especially in countries like Finland with high public oral health coverage for children and adolescents.

Our findings on socioeconomic differences in orthodontic treatment by gender were mixed and require further investigation, for example, whether they stem from differences in orthodontic treatment need, family dynamics, or experience of family socioeconomic status. In this study, there was an equal proportion of boys and girls receiving orthodontic care, but girls seemed to have slightly more orthodontic visits than boys. Gender was not associated with receiving orthodontic care or the number of orthodontic visits when accounting for parental socioeconomic status. Nevertheless, among children and adolescents from lower parental education levels and who received orthodontic care, boys had an average of 10 fewer visits compared to girls. In previous Finnish studies, more girls were in orthodontic treatment (Finnish Institute for Health and Welfare 2024; Nihtilä 2014). There is information on malocclusions by continent, but the differences between girls and boys are limited (Lombardo et al. 2020; Alhammadi et al. 2018; Ghafari et al. 2019). The incidence of malocclusions is population‐specific, although some differences between genders depending on different malocclusions have been noted (Akbari et al. 2016; Eskeli 2015; Ikävalko et al. 2012; Sepp et al. 2017), but not consistently (Sepp et al. 2017). Consequently, as the duration of orthodontic care and the number of visits for different malocclusions vary, it can also affect the number of orthodontic visits between the genders. These differences could be one reason why girls had a higher total number of orthodontic visits over their childhood in the current study.

The strength of our study was the opportunity to make use of register data collected in the PANIC study, a representative population sample, and a long follow‐up period from birth to the age of 16. In addition, even though the data on the use of orthodontic services were manually gathered from the electronic health records, the validity and reliability of these records are very high, as registering those records has been mandatory, and they are also used for dentist remuneration. The weaknesses of our study are that we used only the baseline socioeconomic data when children were between 7 and 9 years of age. It is therefore possible that the socioeconomic status of parents or the child's living conditions, for example, due to the parents' divorce, may have changed during follow‐up. Orthodontic screening visits, provided by orthodontic personnel, could not be reliably distinguished from treatment visits, so total visit counts are likely inflated by a few visits. However, this did not considerably affect the estimated proportion of children receiving orthodontic care, because children with six or more orthodontic visits were classified as having received care, and manual review confirmed these were not merely screening visits.

We did not determine either the actual orthodontic treatments received or the timing of visits during follow‐up, and it is also likely that some participants have received orthodontic care after the end of follow‐up. Even though the PANIC study population reflects the general child population in Kuopio well, the final sample size was relatively small. It is also possible that participants in the PANIC study were more interested in health issues than average, limiting the generalizability to the general population. Like all observational studies, ours is subject to potential unmeasured confounding, such as parental orthodontic history. There may also be some temporal uncertainty regarding the timing of socioeconomic status, orthodontic care, and confounding variables. However, we do not consider this a major issue, as parental socioeconomic position was measured when the child was 7–9 years old, and most orthodontic visits occurred after this age. Additionally, it is unlikely that orthodontic visits would influence parental education, income, or other confounders. Nevertheless, addressing these limitations in future research could enhance the validity and generalizability of the findings and reduce uncertainty around the estimates.

## 5 Conclusions

The findings indicate that socioeconomic status is not likely to have a major impact on receiving orthodontic care or orthodontic visits during childhood in Kuopio, Finland. This finding aligns with the expectations of a health system that adheres closely to the principles of UHC, such as Finland's oral healthcare system for under‐18‐year‐olds, highlighting the potential for equitable healthcare access by socioeconomic status under UHC. There is a need for further research on the topic with population‐wide data to fully understand the dynamics of receiving orthodontic care in socioeconomic groups across different times and places in Finland.

## Author Contributions

**Maria Viisanen:** conceptualization, investigation, formal analysis, writing – original draft. **Eero Raittio:** conceptualization, investigation, formal analysis, supervision, writing – original draft, writing – review and editing. **Tiina Ikävalko:** conceptualization, investigation, supervision, writing – review and editing. **Timo Peltomäki:** conceptualization, supervision, writing – review and editing. **Anna Liisa Suominen:** conceptualization, supervision, writing – review and editing. **Ville Tolonen:** conceptualization, investigation, writing – review and editing. **Sonja Soininen:** conceptualization, investigation, supervision, writing – review and editing. **Henri Karvinen:** conceptualization, investigation, supervision, writing – review and editing. **Timo A. Lakka:** conceptualization, investigation, supervision, resources, funding acquisition, project administration, writing – review and editing.

## Ethics Statement

The study protocol was approved by the Research Ethics Committee of the Hospital District of Northern Savo, Kuopio, Finland.

## Consent

The parents or caregivers of the children gave their written informed consent, and the children provided their assent to participate.

## Conflicts of Interest

The authors declare no conflicts of interest.

## Supporting information


**Figure S1:** Directed acyclic graph with household income as the exposure. Green lines represent the association of interest; red lines indicate confounding pathways; black lines show associations between the outcome and precision variables. **Figure S2:** Directed acyclic graph with parental education as the exposure. Green lines represent the association of interest; red lines indicate confounding pathways; black lines show associations between the outcome and precision variables. **Figure S3:** Interaction between gender and household income or parental education on receiving orthodontic care (A, B), on the total number of orthodontic visits among all participants (C, D) or among those who received orthodontic care (E, F). **Table S1:** Characteristics of participants by gender.

## Data Availability

The data are not publicly available due to research ethical reasons and because the owner of the data is the University of Eastern Finland and not the research group. However, the principal investigator of the PANIC study can provide further information on the study and its data on a reasonable request (timo.lakka@uef.fi).
