# Supplementary material for: Orthodontic Visits According to Socioeconomic Status Among Children Living in the City of Kuopio, Finland: The PANIC Study
Source: Clin Exp Dent Res. 2026 Jan 9;12(1):e70280. doi: 10.1002/cre2.70280 (PMC12784281; doi:10.1002/cre2.70280)
Supplement: Supplementary file 1 — Figure S1: Directed acyclic graph with household income as the exposure. Green lines represent the association of interest; red lines indicate confounding pathways; black lines show associations between the outcome and precision variables. Figure S2: Directed acyclic graph with parental education as the exposure. Green lines represent the association of interest; red lines indicate confounding pathways; black lines show associations between the outcome and precision variables. Figure S3: Interaction between gender and household income or parental education on receiving orthodontic care (A, B), on the total number of orthodontic visits among all participants (C, D) or among those who received orthodontic care (E, F). Table S1: Characteristics of participants by gender. [file CRE2-12-e70280-s001.docx]

**SUPPLEMENTARY MATERIAL**

**Variables which were used in the multiple imputation of missing data:**

Gender; age; Father marital status at baseline; Father marital status at 2-year follow-up; Father marital status at 8-year follow-up; Mother marital status at baseline; Mother marital status at 2-year follow-up; Mother marital status at 8-year follow-up; Mother postal code at baseline; Household income at baseline; Household income at 2-year follow-up; Household income at 8-year follow-up; Parental education at baseline; Parental education at 2-year follow-up; Parental education at 8-year follow-up; Number of orthodontic visits until baseline; number of orthodontic visits until 2-year follow-up; Number of orthodontic visits until 8-year follow-up; Number of dental visits during the follow-up in total; received orthodontic care during the follow-up (over 5 orthodontic visits); Number of orthodontic no-show visits during the follow-up in total; Maternal smoking at baseline; Paternal smoking at baseline; Maternal psychiatric disorder at baseline; Paternal psychiatric disorder at baseline; Maternal toothbrushing frequency at baseline; Paternal toothbrushing frequency at baseline; Maternal alcohol consumption at baseline; Paternal alcohol consumption at baseline; Paternal perceived health; Maternal perceived health; Place of birth; Rye bread consumption; Vegetable and fruit consumption; Margarin consumption; Oil consumption; Fat-free dairy product consumption; Low-fat cheese consumption; Low-fat cold cut consumption; Breakfast habits; Fish consumption; School meal habits; Water drinking; Sugar-sweetened soft drink consumption; Candy consumption; Ice cream consumption; Cookie consumption; Salt consumption; Eating together; Asthma; Allergic rhinitis; Allergic conjunctivitis; Allergic or atopic dermatitis; Lactose-intolerance; Food allergies; Other allergies; Congenital heart disease/defect; Other congenital disease/defect; Attention deficit hyperactivity disorder; Developmental delay; Psychiatric disorder; Visual distortions (eyeglasses); Strabismus; Epilepsy; Diabetes; Juvenile rheumatoid arthritis; Cancer; Coeliac disease; Other disease; Common cold during last 12 months; Bronchitis during last 12 months; Pneumonia during last 12 months; Sinus infection during last 12 months; Pharyngitis during last 12 months; Ear infection during last 12 months; Conjunctivitis during last 12 months; Gastroenteritis or diarrhea during last 12 months; urinary tract infection during last 12 months; Skin infection during last 12 months; Other infection during last 12 months; Antibiotic treatment during last 12 months; Chicken pox; Other pox disease; Weight at birth; Height at birth; Head circumference at birth; Pregnancy duration (weeks); Pregnancy duration (days); Normal pregnancy; Apgar score at 1 minute; Apgar score at 5 minutes; Age at the end of follow-up (31.12.2016).

Table S1. Characteristics of participants by gender.

|  | **Girls, N = 240** | **Boys, N = 264** | **p-value^1^** |
| --- | --- | --- | --- |
| Number of orthodontic visits (mean, SD) | 17.22 (16.07) | 15.05 (15.06) | 0.5 |
| Received orthodontic care |  |  | 0.7 |
| No | 100 (42%) | 114 (43%) |  |
| Yes | 140 (58%) | 150 (57%) |  |
| Age at the baseline (mean, SD) | 7.61 (0.38) | 7.65 (0.40) | 0.5 |
| Age at the end of 2016 (mean, SD) | 15.83 (0.71) | 15.98 (0.72) | 0.043 |
| Household income |  |  | 0.2 |
| Low | 55 (23%) | 51 (20%) |  |
| Medium | 104 (44%) | 100 (39%) |  |
| High | 78 (33%) | 103 (41%) |  |
| Unknown | 3 | 10 |  |
| Parental education |  |  | 0.009 |
| Low | 44 (18%) | 54 (21%) |  |
| Medium | 123 (51%) | 99 (38%) |  |
| High | 72 (30%) | 106 (41%) |  |
| Unknown | 1 | 5 |  |
| Mother’s marital status |  |  | 0.7 |
| Married/cohabitation | 194 (82%) | 218 (85%) |  |
| Single | 16 (6.7%) | 14 (5.5%) |  |
| Separated/divorced | 27 (11%) | 22 (8.6%) |  |
| Widow | 1 (0.4%) | 1 (0.4%) |  |
| Unknown | 2 | 9 |  |
| Parental self-rated health |  |  | 0.7 |
| Excellent | 17 (7.4%) | 21 (8.5%) |  |
| Very good | 97 (42%) | 109 (44%) |  |
| Good | 86 (38%) | 88 (36%) |  |
| Poor | 28 (12%) | 25 (10%) |  |
| Very poor | 1 (0.4%) | 4 (1.6%) |  |
| Unknown | 11 | 17 |  |
| Parental psychiatric disorder |  |  | >0.9 |
| Yes | 42 (18%) | 46 (18%) |  |
| No | 197 (82%) | 210 (82%) |  |
| Unknown | 1 | 8 |  |
|  | | | |
| ^1^Wilcoxon rank sum test; Pearson's Chi-squared test; Fisher's exact test | | | |


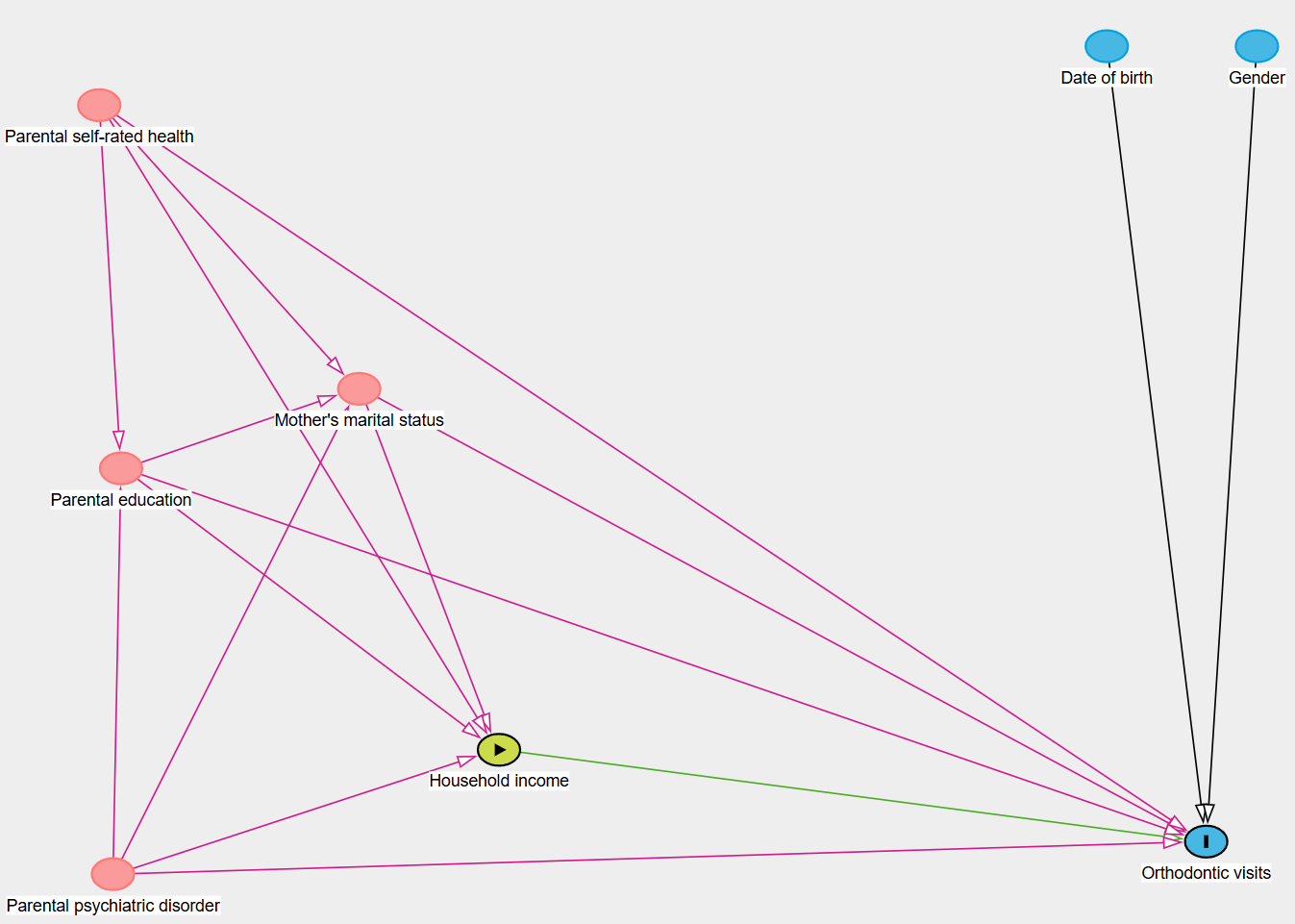


Figure S1. Directed acyclic graph with household income as the exposure. Green lines represent the association of interest; red lines indicate confounding pathways; black lines show associations between the outcome and precision variables.


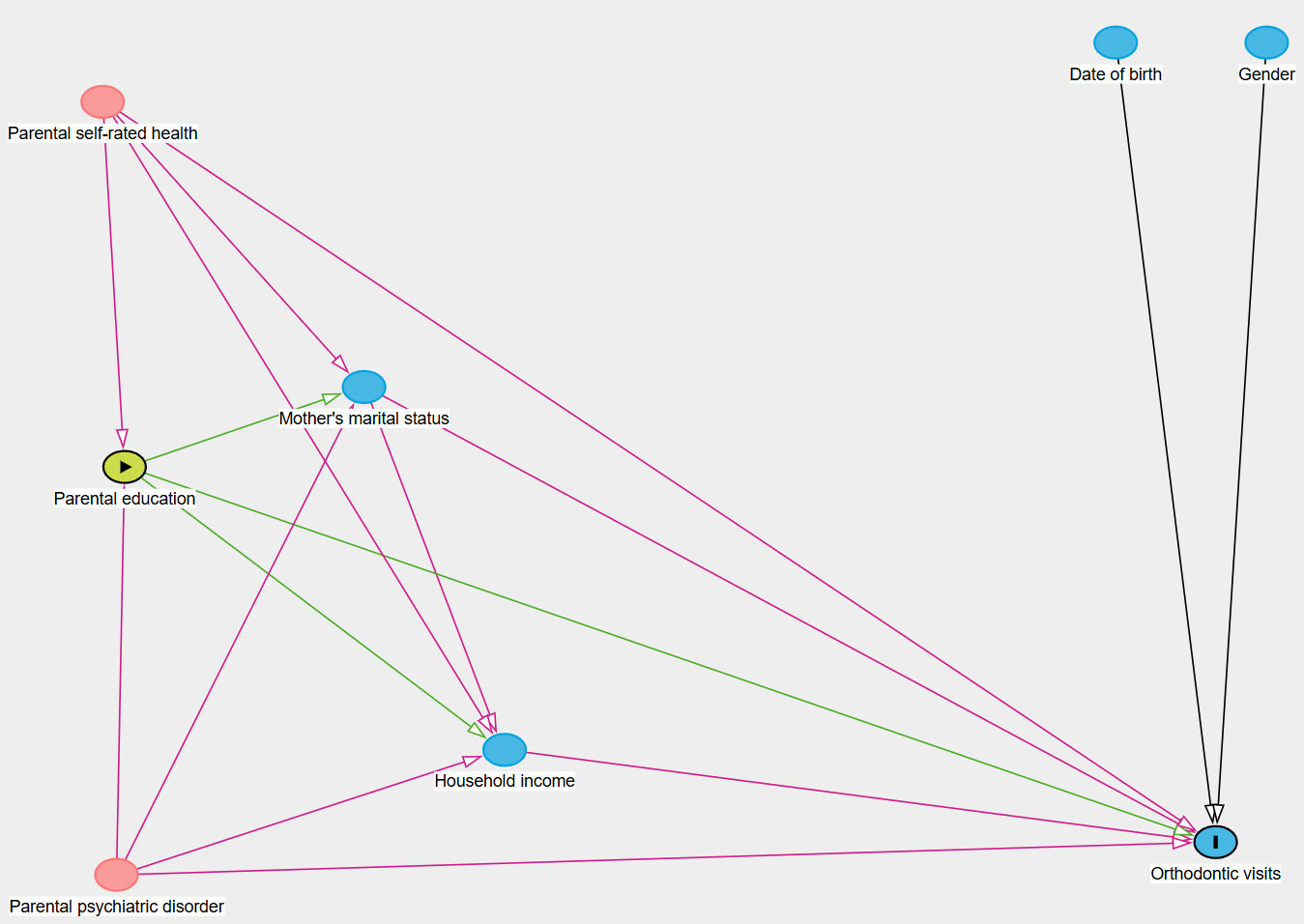


Figure S2. Directed acyclic graph with parental education as the exposure. Green lines represent the association of interest; red lines indicate confounding pathways; black lines show associations between the outcome and precision variables.

**
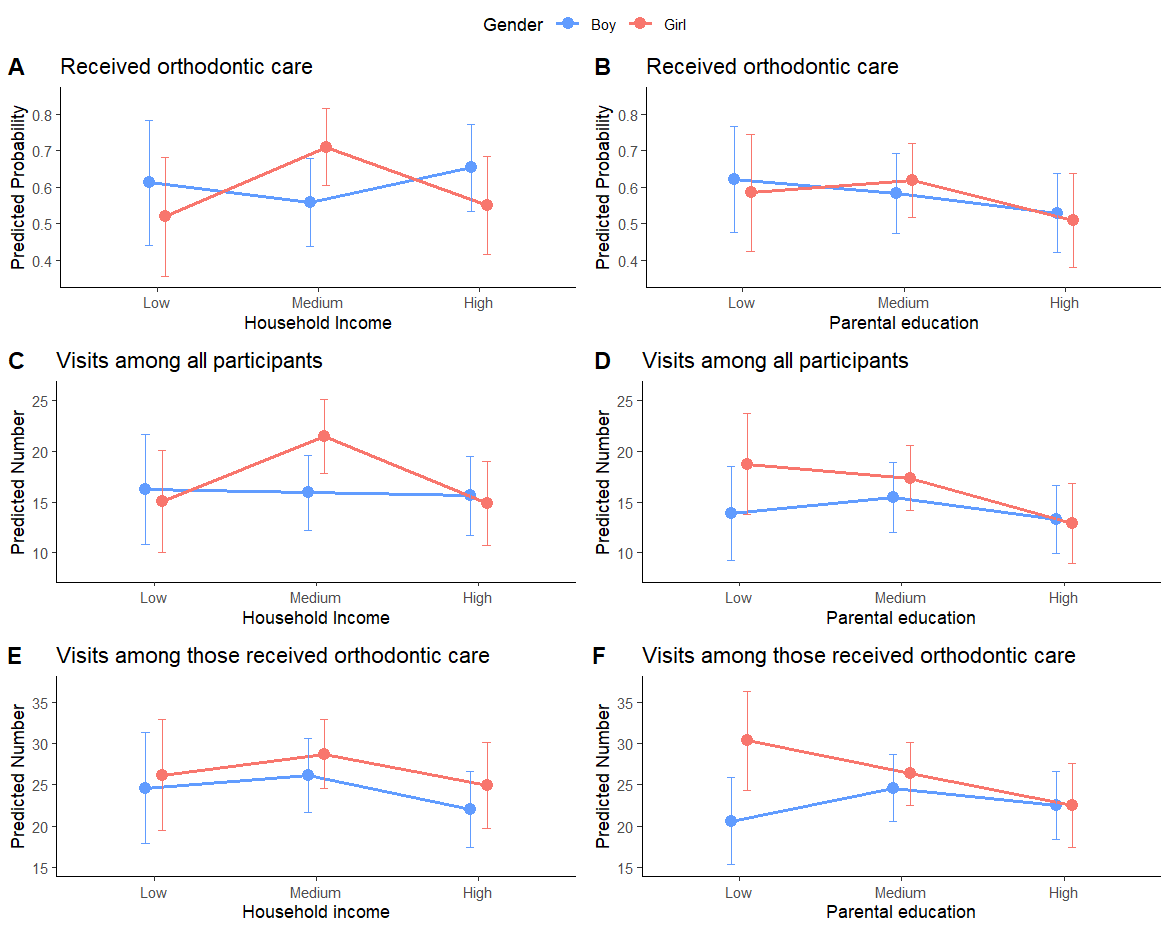
**

Figure S3. Interaction between gender and household income or parental education on receiving orthodontic care (A, B), on the total number of orthodontic visits among all participants (C, D) or among those who received orthodontic care (E, F).
